# A Case Report of an Intrathoracic Mass Lesion Caused by *Arcanobacterium haemolyticum* that Required Exclusion of a Malignant Tumor Diagnosis

**DOI:** 10.31662/jmaj.2018-0036

**Published:** 2019-05-16

**Authors:** Yusuke Seki, Daiichi Morii, Kazunori Hata, Toshiyuki Unno, Takayuki Yokozawa, Sayori Li, Toshimi Oda

**Affiliations:** 1 Department of Infectious Diseases, Showa General Hospital, Tokyo, Japan; 2 Department of Infection Control and Prevention, Graduate School of Medicine, Osaka University, Osaka, Japan; 3 Department of Thoracic Surgery, Showa General Hospital, Tokyo, Japan; 4 Department of Radiology, Showa General Hospital, Tokyo, Japan; 5 Department of Clinical Laboratory, Showa General Hospital, Tokyo, Japan; 6 Department of Orthopedic Surgery, Showa General Hospital, Tokyo, Japan

**Keywords:** *Arcanobacterium haemolyticum*, intrathoracic mass lesion, diabetes mellitus, compromised host

## Abstract

A 57-year-old man with untreated diabetes mellitus was admitted to our hospital due to an intrathoracic mass lesion infiltrating the vertebral body and mediastinum. The mass was suspected to be invasive lung cancer; however, percutaneous needle biopsy revealed that the mass was inflammatory granulation tissue caused by an *Arcanobacterium haemolyticum* infection. To the best of our knowledge, this is the first report of an intrathoracic mass lesion caused by an *A. haemolyticum* infection. When an intrathoracic mass lesion is suspected, clinicians should consider possible infections that cause granulation tissue, such as *A. haemolyticum*. This is particularly important in immunocompromized hosts such as patients with diabetes.

## Introduction

*Arcanobacterium haemolyticum* (formerly *Corynebacterium haemolyticum*) was first described in pharyngitis and skin lesions by MacLean in 1946 ^(1), (2)^, and it is a β-hemolytic, gram-positive, rod-shaped, and catalase-negative bacillus ^(1), (2)^. Although *A. haemolyticum* is regarded as part of the throat and skin normal flora ^(3)^, this is the first known report of granulation tissue in the thorax that infiltrated the pleural cavity and mediastinum with purulent spondylitis and bacteremia ^(3), (4), (5), (6)^. This case highlights the importance of bacteriological examination as an essential step in excluding malignancy.

## Case Report

**Figure 1.**
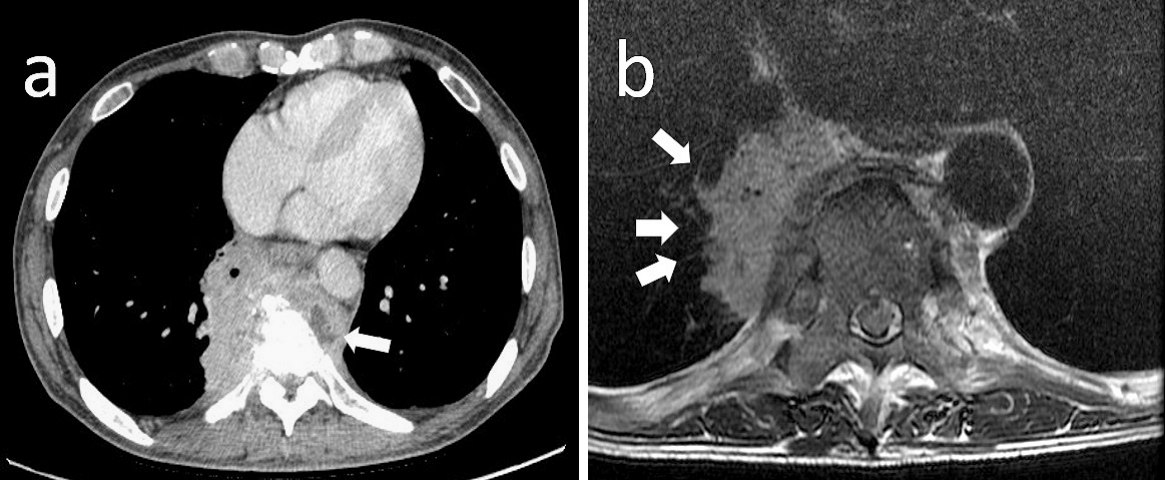
Imaging studies on admission. (a) Contrast-enhanced computed tomography (CT) on admission demonstrated an invasive mass lesion with non-homogenous enhancement. The mass measured 85 × 67 mm and was located adjacent to S7 of the right lung, with bone infiltration and a compression fracture of the thoracic vertebrae. A low-density area (white arrow) was partially visible on the left side of the mass. (b) A magnetic resonance (MR) T2-weighted image showed that the mass spiraled up the pulmonary vessels (white arrows) and markedly infiltrated the thoracic vertebrae.

A 57-year-old man with no past medical history visited a local clinic for right hypochondralgia and unintentional weight loss (20-kg loss in 3 months). Initially, he was diagnosed as having diabetes mellitus with suspected cholelithiasis. The diabetes mellitus had not been treated and the status of glycemic control had been estimated to be poorly controlled. Three months later, the pain radiated to his back, which aggravated while walking. Chest computed tomography (CT) showed an invasive intrathoracic mass lesion measuring 72 × 30 mm, which seemed to compress the lower right lung lobe from the mediastinum and partially infiltrated the thoracic vertebrae T7 – T8, which were adjacent to the mass shadow and partially osteolytic. Whether the mass was derived from the pulmonary parenchyma, pleura, mediastinum, or thoracic vertebrae remained unclear. The patient was referred to our hospital for needle biopsy. His vital signs on admission were a Glasgow Coma Scale score of E4V5M6, blood pressure of 133/90 mmHg, respiratory rate of 14 breaths/min, body temperature of 38.7℃, and pulse rate of 98 beats/min. Neurological examination showed no significant findings. The number of leukocytes was 9.7 × 10^9^/L and the C-reactive protein level was 11.69 mg/dL. Hemoglobin was 12.2 g/dL, the number of erythrocytes was 3.95 × 10^6^/L, and hematocrit was 36.5%. Hemoglobin A1c was 6.7%. Any tumor marker levels were not elevated. The HIV test was negative. Three sputum acid-fast bacteria smear tests were negative. Enhanced CT revealed that the invasive mass increased to 85 × 67 mm in 4 weeks. The mass passed through the bronchus and infiltrated the pulmonary parenchyma, with bone infiltration and compression fracture of the thoracic vertebrae (Figure 1a). A low-density area was considered to be necrosis caused by a malignant tumor or an abscess owing to infection. The diffusion-weighted image on magnetic resonance imaging showed no high signal intensity typical of an abscess (Figure 1b). As the mass was slowly progressive, differential diagnosis also included actinomycetes, fungi, or non-tuberculous mycobacterium as atypical infections. However, lung cancer was more likely suspected than these infections owing to the involvement of the bronchus and pulmonary vessels and the location in the lung, vertebral body, and mediastinum.

**Figure 2.**
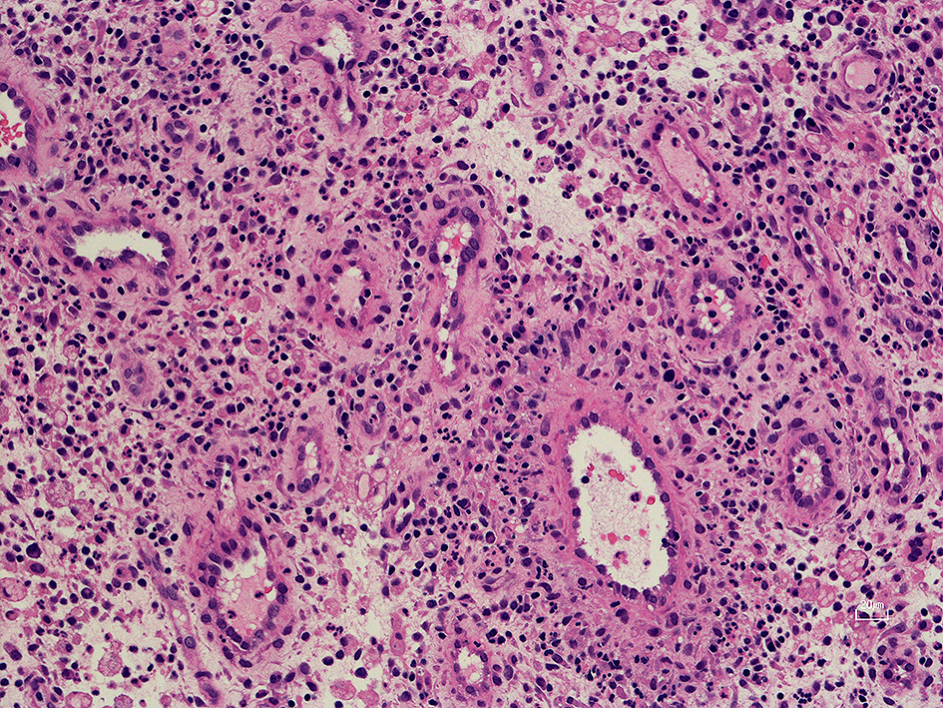
Biopsy findings. A computed tomography-guided biopsy specimen demonstrated inflammatory granulation by neutrophils, histiocytes, plasma cells, and lymphocytes, which was nonspecific inflammation in the subacute and chronic phase. Hematoxylin & eosin stain, magnification × 50.

**Figure 3.**
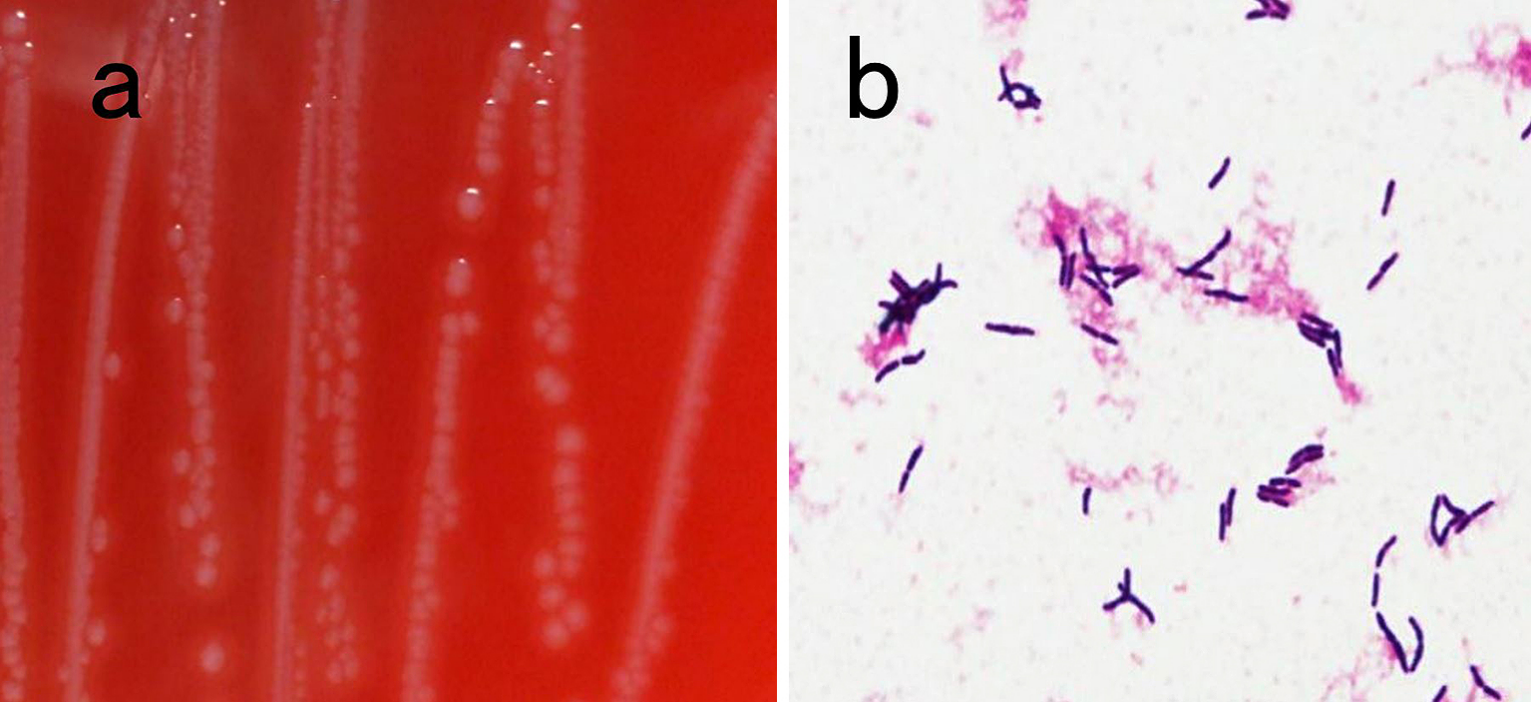
Results of tissue and blood cultures. (a) β-hemolysis on sheep blood agar; (b) Gram stain of *Arcanobacterium haemolyticum* in blood culture.

On hospital day 4, CT-guided percutaneous needle biopsy was performed. Histological examination of the right lung specimen revealed inflammatory granulation tissue, fibrous tissue, and no evidence of malignancy (Figure 2). A tissue culture was performed and bacterial colonies of around 1-mm diameter were found and showed β-hemolysis on sheep blood agar after a 48-h culture (Figure 3a). The specimen culture isolated gram-positive rods. Two sets of blood cultures were obtained: on admission and hospital day 8; these cultures also revealed gram-positive rods (Figure 3b). The BD Phoenix system and MALDI-TOF MS identified *A. haemolyticum*. The strain was susceptible to most antimicrobials, including penicillin and cephalosporins ^(6), (7)^. Intravenous ampicillin/sulbactam was selected based on a susceptibility test (Table 1). On hospital day 9, the patient had difficulty in raising both legs, and neurological examination revealed bladder rectum disorder and decreased sensation under T9. We transferred the patient to another specialty hospital for emergency surgery for spinal decompression. Although inferior spinal fusion was performed, the bladder rectum disorder continued.

**Table 1.** Results of Antimicrobial Susceptibility Testing.

Antimicrobial agent	MIC (µg/mL)	Sensitivity
Benzylpenicillin (PCG)	≤1	S
Ceftriaxone (CTRX)	≤0.5	S
Cefepime (CFPM)	≤0.5	S
Imipenem/Cilastatin (IPM/CS)	≤0.25	S
Gentamicin (GM)	≤4	S
Erythromycin (EM)	≤0.5	S
Clindamycin (CLDM)	≤0.5	S
Minocycline (MINO)	≤2	S
Levofloxacin (LVFX)	≤2	S

Abbreviations: MIC, minimum inhibitory concentration; S, susceptible

## Discussion

Since 1946, several case reports showed that *A. haemolyticum* can cause brain abscesses, liver abscesses, thyroid abscesses, infective endocarditis, skin and soft tissue infections, osteomyelitis, and bacteremia, especially in patients who are immunocompromized ^(1), (3), (4), (5), (6)^.

In our case, the invasive mass was highly suspected as lung cancer infiltrating the thoracic vertebrae. Although our differential diagnosis included infections ^(7), (8)^, we could not identify a published case report where *A. haemolyticum* caused inflammatory granulation tissue that infiltrated the thoracic vertebrae.

Untreated diabetes mellitus might have played a role in *A. haemolyticum* infection development in our patient. Most previous case reports of *A. haemolyticum* infections reported patients who were immunodeficient, which is a state commonly associated with malignant tumors and diabetes mellitus ^(3), (4), (5), (7)^.

In our case, in spite of the slightly high Hemoglobin A1c level, the patient unintentionally lost 20 kg in 3 months, implying that a severe immunodeficiency may have been caused by diabetes mellitus. Such a condition continued for a long time, and the *A. haemolyticum* infection progressed invasively.

In conclusion, when an intrathoracic mass suspected to be a malignant tumor is observed on imaging, clinicians should consider infections causing inflammatory granulation tissue such as an *A. haemolyticum* infection. This is particularly important in immunocompromized hosts, including patients with diabetes mellitus.

## Article Information

### Conflicts of Interest

None

### Acknowledgement

We thank Editage for their English editorial assistance.

### Author Statement

All authors meet the ICMJE authorship criteria.

### Consent for Publication

Written informed consent was obtained from the patient for publication of this case report and any accompanying images.
